# Host plasticity supports spread of an aquaculture introduced virus to an ecosystem engineer

**DOI:** 10.1186/s13071-020-04373-y

**Published:** 2020-10-01

**Authors:** Babette Bookelaar, Sharon A. Lynch, Sarah C. Culloty

**Affiliations:** 1 School of Biological, Earth and Environmental Sciences, University College Cork, Cork, Ireland; 2 Aquaculture and Fisheries Development Centre and Environmental Research Institute, University College Cork, Cork, Ireland; 3 MaREI Centre, Environmental Research Institute, University College Cork, Cork, Ireland

**Keywords:** Ostreid herpes virus-1 microVar, Trophic, Viral transmission dynamics, Ecosystem engineer, Species jump, Pathogen-host-environment interplay, *Crassostrea gigas*, *Cerastoderma edule*

## Abstract

**Background:**

The common cockle *Cerastoderma edule* plays an important ecological role in the marine ecosystem both as an infaunal engineer (reef forming and bioturbation) and a food source for protected bird species in its European range. Cockle beds are found in close proximity to aquaculture and fisheries operations, which can be “hot spots” for infectious agents including viruses and bacteria. Ostreid herpesvirus-1 microVar (OsHV-1 μVar) has spread to many Pacific oyster *Crassostrea gigas* culture sites globally, where it has been associated with significant mortalities in this cultured bivalve. Knowledge on the impact of the virus on the wider ecosystem, is limited. As the likelihood of released virus dispersing into the wider aquatic ecosystem is high, the plasticity of the virus and the susceptibility of *C. edule* to act as hosts or carriers is unknown.

**Methods:**

In this study, wild *C. edule* were sampled biweekly at two *C. gigas* culture sites over a four-month period during the summer when OsHV-1 μVar prevalence is at its highest in oysters. *C. edule* were screened for the virus molecularly (PCR, qPCR and Sanger sequencing) and visually (*in situ* hybridisation (ISH)). The cockle’s ability to act as a carrier and transmit OsHV-1 μVar to the oyster host at a temperature of 14 ℃, when the virus is considered to be dormant until water temperatures exceed 16 ℃, was also assessed in laboratory transmission trials.

**Results:**

The results demonstrated that OsHV-1 μVar was detected in all *C. edule* size/age cohorts, at both culture sites. In the laboratory, viral transmission was effected from cockles to naïve oysters for the first time, five days post-exposure. The laboratory study also demonstrated that OsHV-1 μVar was active and was successfully transmitted from the *C. edule* at lower temperatures.

**Conclusions:**

This study demonstrates that OsHV-1 μVar has the plasticity to infect the keystone species *C. edule* and highlights the possible trophic transmission of the virus from cockles to their mobile top predators. This scenario would have important implications, as a greater geographical range expansion of this significant pathogen *via* migratory bird species may have an impact on other species that reside in bird habitats most of which are special areas of conservation. 
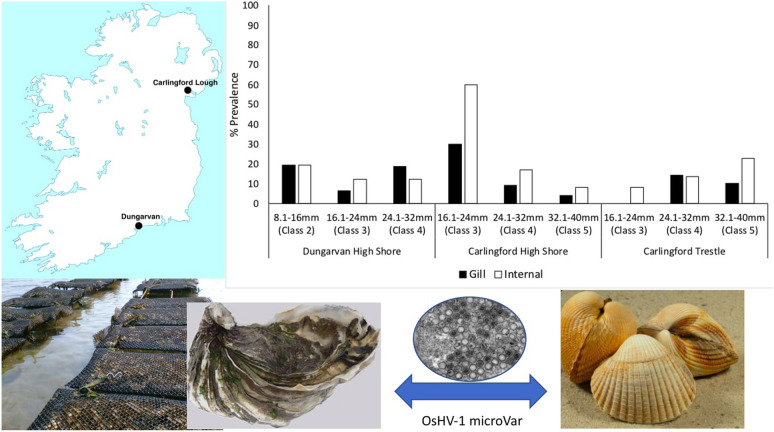

## Background

Coastal and marine ecosystems are routinely used for fisheries and aquaculture purposes [1–3] with culture sites often being located on intertidal mud and sandbanks, which have a diverse community of marine invertebrates, algae and vertebrates, including protected wading bird species [4]. Such habitats support a complex interwoven food web with a number of trophic levels. Pathogens and diseases are common in marine habitats [5], often as a result of a complex interplay between the natural host, its environment and the pathogen [6], which can have a significant impact on fisheries and aquaculture due to associated intensive culture [5, 7].

*Cerastoderma edule* is widely distributed in intertidal ecosystems in North Africa and Europe [8, 9] and is of commercial importance particularly in the British Isles, The Netherlands, Spain and France [8–11]. Moreover, *C. edule* plays a significant ecological role as an ecosystem engineer [8, 12, 13], by directly and/or indirectly altering the marine ecosystem. Using its physical structure, *C. edule* can directly shape its marine environment by forming reefs [13, 14] and as bioturbators it can mobilize sediments and affect sediment stability and accumulation [13, 15–17]. *Cerastoderma edule* functions as a link between primary producers by being a suspension feeder and therefore indirectly change levels of algae, nutrients/metals and sediments [17–20]. It can also indirectly affect higher trophic levels [20, 21] by acting as an important food source for cohabiting marine species such as crustaceans (the shore crab *Carcinus maenas* [22] and brown shrimp *Crangon crangon* [23]), fish species (the European plaice *Pleuronectes platessa* [24]) and protected wading bird species such as the curlew *Numenius arquata* and the oystercatcher *Haematopus ostralegus* [25].

Cockle mortalities have been reported sporadically [26–30] caused by predation, unfavourable environmental circumstances, overfishing, but have also been linked with pathogens and diseases [31]. *Cerastoderma edule* provides a habitat to a wide range of pathogens and diseases including viruses, bacteria, fungi (Microsporidia), Apicomplexa, Amoeba, Ciliophora, Perkinsozoa, Haplosporidia, Cercozoa, Turbellaria, Digenea, Cestoda, Nematoda, Crustacea and Nemertea [8].

One important global aquaculture bivalve species is the Pacific oyster *Crassostrea gigas* [32] that cockles often share intertidal beds with. *Crassostrea gigas* culture has been affected by significant mortalities, associated with ostreid herpesvirus-1 (OsHV-1) and its variants including ostreid herpesvirus-1 microVar (OsHV-1 μVar) since the 1990’s [33–35]. Past studies have investigated the possibility of other marine invertebrates being carriers and or reservoirs of OsHV-1 and OsHV-1 μVar, outside the known host *C. gigas.* A herpes-type infection, the first to be detected in an invertebrate, was observed in the eastern oyster *Crassostrea virginica* experimentally [36]. In that study, a higher prevalence of the virus occurred in oysters when seawater temperatures were elevated above the normal ambient temperature. A variant of OsHV-1 has been associated with high mortalities of cultured Farrer’s scallops *Chlamys farreri* in China [37]. López Sanmartín et al. [38] demonstrated that the European flat oyster *Ostrea edulis* can be infected by OsHV-1 μVar when the virus is administered as an intramuscular injection. More recently OsHV-1 μVar was detected in different marine invertebrates including the Sydney cockle *Anadara trapezia* in Australia [39], in cultured *Mytilus galloprovinciallis* in Italy [40], in wild *Mytilus spp*. consisting of the blue *Mytilus edulis*, *M. galloprovinciallis* and hybrids of both parent species, in Ireland [41] and the green shore crab *C. maenas* [42]. Bookelaar et al. [42] demonstrated that transmission of the virus could occur from *C. maenas*, previously exposed to the OsHV-1 μVar in the wild, to cohabiting naïve *C. gigas* within four days. These findings provided some insight on the potential for pathogen species to jump host and more importantly the possibility that this virus can be transmitted through marine food webs and dispersed over greater distances *via* mobile top predators.

As the cockle plays a significant ecological role, this study investigated the potential of this keystone species as a host, carrier or reservoir of infection for this significant pathogen. The ultimate focus of this study was to shed new light on the potential impacts of anthropogenic introduced pathogens in a wider ecological context.

## Methods

The study was conducted in two parts; the first was a field study to screen cockles from areas where oyster culture was taking place for the presence of OsHV-1 μVar and the second part was a laboratory transmission trial to determine if virus could be detected in *C. edule* from an OsHV-1 μVar endemic Pacific oyster culture site and if transmission could occur to naïve *C. gigas*.

### Field study sites

**Fig. 1:**
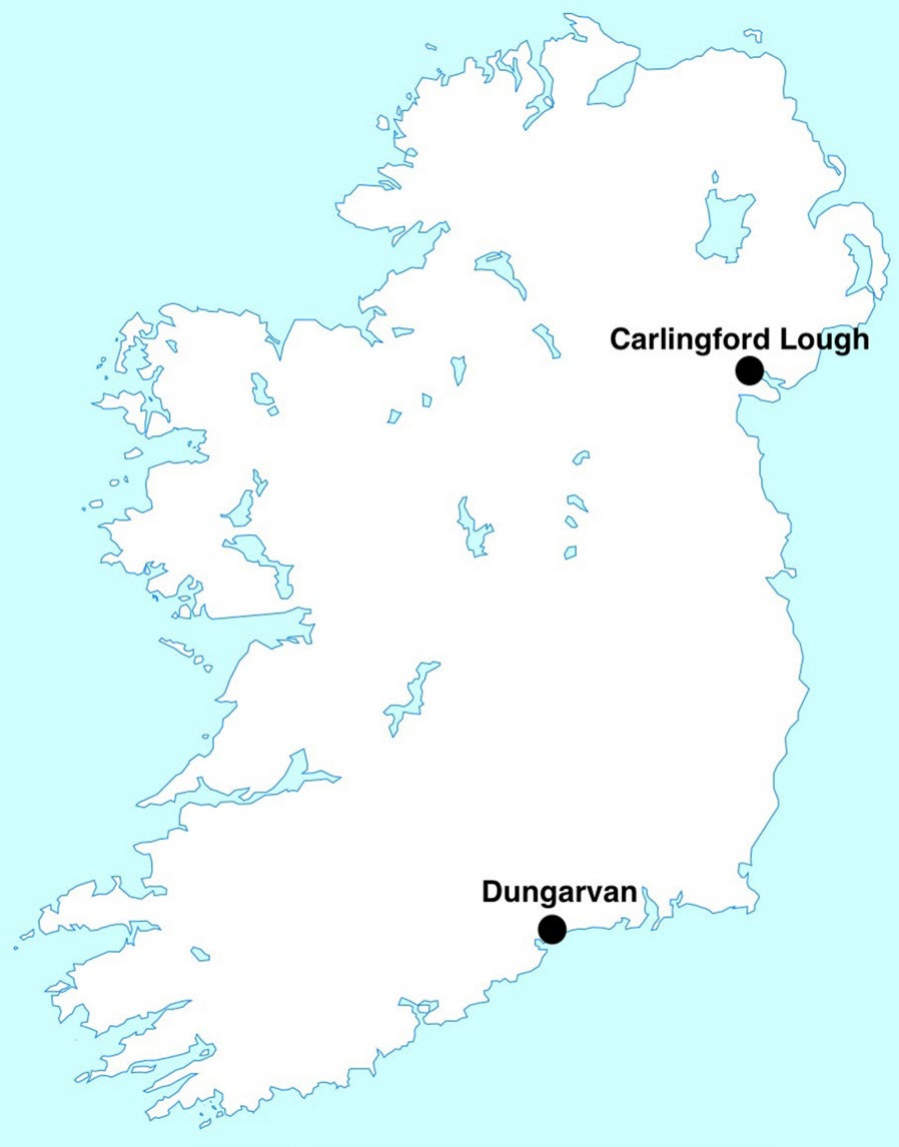
Oyster *Crassostrea gigas* culture sites in Ireland where *Cerastoderma edule* were sampled

This study took place at the two main Irish oyster culture sites; Dungarvan, Co. Waterford (52.0704°N, 7.5939°W), which covers an area of approximately 25 km^2^ and Carlingford Lough, Co. Louth (54.0733°N, 6.1994°W), which covers an area of approximately 50 km^2^ [42] (Fig. 1) [42–44]. For both culture sites OsHV-1 μVar has been present since 2009 [45].

In Ireland, oyster culture sites including Dungarvan and Carlingford Lough are known as Special Protection Areas (SPA) under the EU Birds and Habitats Directives as they support over 20,000 waterbirds during the non-breeding season and are of specific conservation interest and importance for bird species [43, 44]. Mobile predators like birds (the oystercatcher *Haematopus ostralegus* and the common tern *Sterna hirundo*) are observed in Irish coastal areas year round and are known to prey on *C. edule* 10–30 mm in shell length, on the upper shore [46].

### Environmental data

Environmental data loggers (Star-Oddi) supplied by Bord Iascaigh Mhara (BIM) were used to measure and record continuous water temperature values (every half hour) from the end of May until mid-August 2015 for both sites. Due to a technical issue, data were not recorded from the end of June to the end of July at Dungarvan.

### Bivalve sampling

In this study, surfaced (gaping and non-gaping) cockles were randomly sampled at both *C. gigas* culture sites. Taking into consideration the potential extension range of the virus in the intertidal zone (e.g. by transport of infected particles through the water column), cockles were sampled around the oyster trestles (main culture site) during low tide and approximately 500-m higher up the shore. Thirty cockles were taken at the high shore at both sites and at the oyster trestles in Carlingford Lough (as no surfaced cockles were observed at the trestles in Dungarvan). In addition, to detect baseline levels of virus in the natural host, at every sampling date, 30 *C. gigas*, originally imported from French hatcheries, were collected at the oyster trestles at both sites.

Cockles were collected biweekly nine times in Dungarvan (April 2015 to August 2015) and eight times in Carlingford Lough (April 2015 to August 2015). As cockles were not present at the trestles in Dungarvan, cockles (250 individuals in total) were only collected at the high shore. At Carlingford Lough, 445 cockles were collected for screening, 218 cockles at the high shore and 227 cockles at the trestles.

Cockles and oysters were processed for histology and molecular analysis on return to the laboratory.

### Laboratory transmission trial

Naïve *C. gigas* (*n* = 150, average weight of 3.0 g and average length of 31.3 mm), which had never been exposed to OsHV-1 μVar, were obtained from an Irish hatchery in October 2015. *Crassostrea edule* (*n* = 93, average weight of 12.6 g and average shell length of 29.9 mm) were randomly collected in October 2015 from Carlingford Lough, where OsHV-1 μVar had been detected in oysters. Prior to the start of the trial, 30 naïve *C. gigas* and 30 *C. edule* were screened for OsHV-1 μVar by polymerase chain reaction (PCR), to confirm the oysters were uninfected and to determine if the virus could be detected in *C. edule*.

Before placing in tanks, the exterior of the shells of *C. edule* were washed several times in ddH_2_O to remove any pathogens that may have been incidentally attached to their external shell. Four 10-l tanks were filled with 8-l UV filtered natural seawater and animals were held at 14 ℃ in a constant temperature (CT) room with a salinity of 35. The experimental set up consisted of a control tank containing 30 naïve oysters and three replicate experimental tanks, which each contained 30 naïve *C. gigas* and 21 *C. edule*. The tanks were checked several times daily for mortality and any gaping and/or dead individuals were removed to screen for OsHV-1 μVar by PCR. In addition, oysters (*n* = 3) were sampled on Day 2 (48 hours), Day 4 (96 hours) and Day 7 (168 hours), to screen the naïve oysters for the virus to determine if any replication of the virus was taking place by assessing viral loads. All individuals, *C. gigas* and *C. edule*, still alive at the end of the experiment were removed and screened for OsHV-1 μVar. Since most cockles died within the first few days the trial was terminated at 14 days.

### Cockle sample processing

Whole wet weight (g) and shell length (mm) of cockles from both field and laboratory trials were recorded using a balance scales and Vernier calipers. In addition, shell rings were counted. Shell lengths were divided into five different length classes: Class 1: 0–8 mm; Class 2: 8.01–16.00 mm; Class 3: 16.01–24.00 mm; Class 4: 24.01–32.00 mm; and Class 5: 32.01–40.00 mm. Weights were categorized as four weight classes: Class 1: 0–7 g; Class 2: 7.01–14.00 g; Class 3: 14.01–21.00 g; and Class 4: 21.01–28.00 g. Growth rings on the shell were divided into seven different shell ring classes: Class 1: 0 rings; Class 2: 1 ring; Class 3: 2 rings; Class 4: 3 rings; Class 5: 4 rings; Class 6: 5 rings; and Class 7 > 5 rings. Correlation tests were performed between weight and age and between shell length and age to clarify if growth rings could be associated with life span.

### DNA extraction

Gill and internal tissues (approx. 5 mm^2^) made up of connective, digestive and reproductive tissues of *C. edule* and gill tissue of *C. gigas*, from both field and laboratory trials, were stored in 95% ethanol for DNA isolation to detect OsHV-1 μVar. Before DNA extraction took place, tissues were washed in double deionized water (ddH_2_O) and blot dried using tissue paper. DNA extraction was performed using the Chelex-100 methodology [47]. Tissue samples were placed in a 10% chelex solution (100 µl volume) (Sigma Aldrich) and following the samples were placed in a Hybaid thermal cycler for 1 h and 10 min heated at 99 ℃ to facilitate cell lysis [47]. A subsample (*n* = 30) of the cockle DNA was quantified by a spectrophotometer (NanoDrop 1000 spectrophotometer) to confirm DNA quantity and quality to avoid the diagnosis of false negatives due to low DNA quantity and/or DNA of poor quality. The ratio of absorbance at 260 nm and 280 nm was used to assess the purity of DNA with a ratio of ~ 1.8 considered “pure” for DNA.

### Polymerase chain reaction (PCR)

Standard PCR to detect OsHV-1 μVar was performed using the OHVA/OHVB primers [48]. Positive controls (duplicate) consisting of OsHV-1 μVar infected oyster tissue and negative controls (duplicate) of double distilled water (ddH_2_O) were used in duplicate for each PCR. Agarose gel electrophoresis was carried out using a 2% agarose gel stained with ethidium bromide (10 mg/l stock) and was run with an electrical charge of 110V for 45–60 min. Expected size of amplified PCR products for OsHV-1 μVar was 385 bp [48].

### Quantitative polymerase chain reaction (qPCR)

Quantitative PCR (qPCR) was carried out to determine the viral load of a subsample of cockles from the field trial (*n* = 76), including gill (*n* = 33) and internal tissue (*n* = 43) of cockles from both sites, deemed positive for OsHV-1 μVar by standard PCR. In addition, qPCR was performed on *C. edule* and *C. gigas* from the laboratory transmission trial, which were deemed positive for OsHV-1 μVar by standard PCR. qPCR conditions, master mix and thermocycling conditions were carried out using the HVDP-F and HVDP-R primers [49]. Samples were tested in duplicate and samples with a mean CT value of below 37 were indicative of a positive result for the virus.

### *In situ* hybridization (ISH)

*In situ* hybridization assays (ISH) were carried out on cockles from the field survey that were negative (*n* = 3 for both sites) and positive (*n* = 3 for both sites) for OsHV-1 μVar when screened by PCR and qPCR. In addition, ISH was performed on infected (*n* = 3) and uninfected (*n* = 3) *C. gigas* from the laboratory trial. An ISH protocol that has been used to screen for *Bonamia ostreae* was carried out using a digoxigenin (DIG)-labelled probe specific to OsHV-1 μVar [50].

### Sanger sequencing

Sequencing of genomic DNA from positive PCR products amplified from *C. edule* (*n* = 5) from the field screening (Carlingford (*n* = 3) and Dungarvan (*n* = 2)) took place. PCR products in triplicate from each individual were pooled together to increase DNA concentration prior to being sent for direct sequencing to Eurofins MWG. DNA sequences that were generated by Eurofins MWG were matched against the National Center for Biotechnology Information (NCBI) nucleotide database with BLASTn to identify and confirm that the sequences were specifically OsHV-1 μVar strains.

### Statistical analyses

Statistical analyses were performed in statistical model program R studio [51]. Normality was tested using the Shapiro-Wilks normality test. Environmental data between sites were analysed by Studentʼs t-test. Morphometrics (length and weight) between sites and shore heights were analysed by Mann-Whitney U-test. Spearmanʼs rank order correlation was used to determine if there was an association between shell rings and weight and between shell rings and length. Pearsonʼs Chi-square tests were used to compare length and weight classes and number of shell rings between sites and within sites at the two shore heights. Pearsonʼs Chi-square tests were also used to test for differences of prevalence of OsHV-1 μVar for gill and internal tissue for cockle within sites at the two shore heights. For all analyses, a critical value of 0.05 was used to confirm significant results. Data are presented as the mean ± standard error.

## Results

### Baseline levels of OsHV-1 μVar at Pacific oyster culture sites

**Fig. 2:**
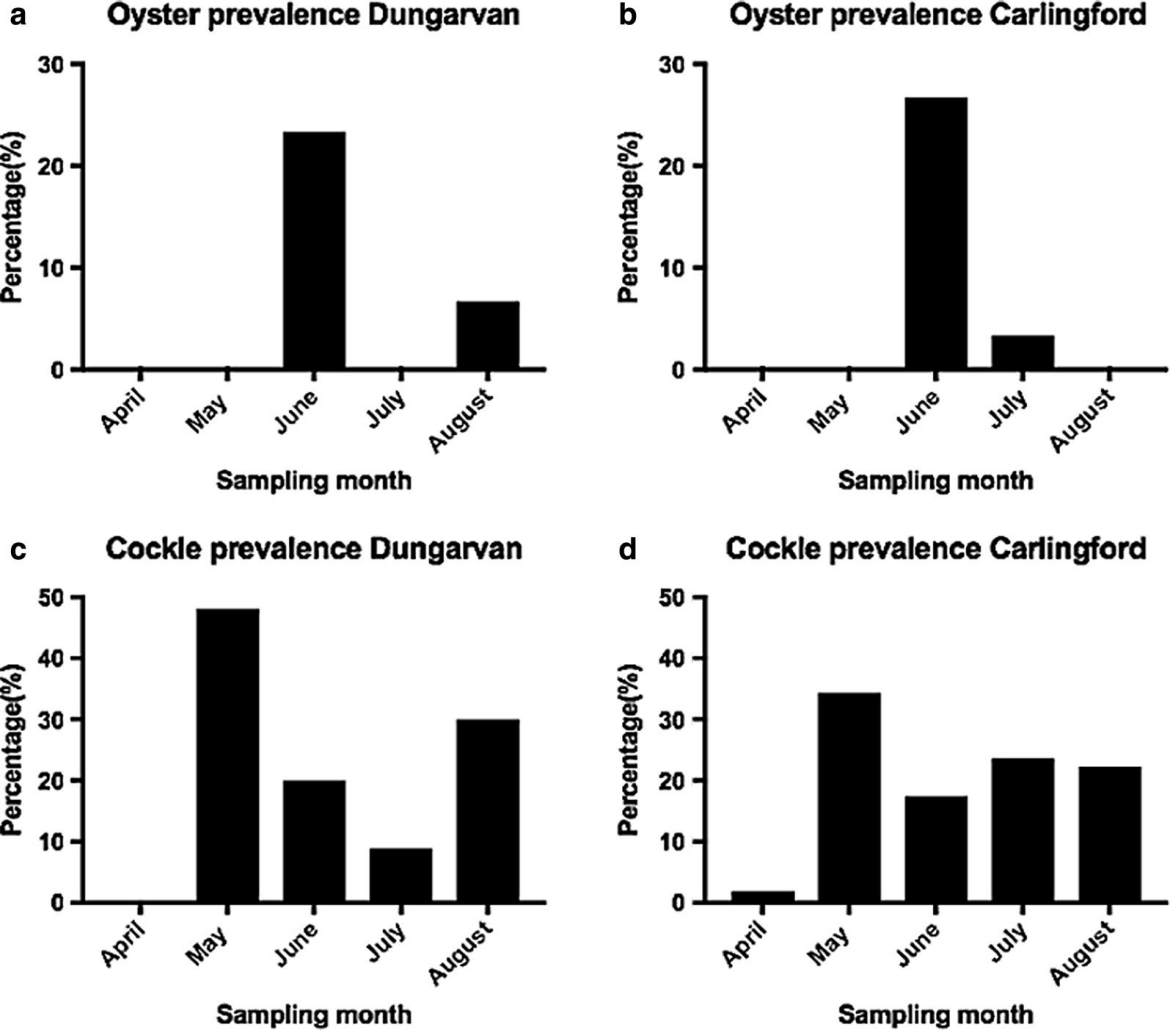
OsHV-1 μVar prevalence (%) in oysters (**a**, **b**) and cockles (**c**, **d**) at the sites

OsHV-1 μVar was present in oysters at the sample sites. Overall prevalence of OsHV-1 μVar by PCR in *C. gigas* at Dungarvan (*n* = 270) and Carlingford Lough (*n* = 240) for the duration of the field trial was low (< 10%) with a mean prevalence of 6.0% and a range of 0–27% per month at Dungarvan and a mean prevalence of 6.0% with a range of 0–23% per month at Carlingford Lough (Fig. 2). The overall water temperature during summer 2015 remained low (< 16 ℃).

### Virus detection in *Cerastoderma edule* in the field

OsHV-1 μVar was detected in the cockles at both sample sites, and in both gill and internal tissues in the PCR screening. A subsample of cockle DNA (*n* = 30) was screened for DNA quantity and quality using a NanoDrop spectrophotometer. Regarding quantity all samples showed a sufficient amount of DNA higher than 40 μg/l and 80% of the samples had DNA higher than 100 μg/l. 260/280 ratio indicated values of 1.5–2.1. 260/230 ratios represented values ranging between 1.5 and 2.0, representing low contamination levels.

The overall prevalence of OsHV-1 μVar in *C. edule* for Dungarvan was 14.4% (14.4% at high shore) and in Carlingford Lough was 13.6% (12.8% at high shore and 14.3% at trestles). More individuals demonstrated the presence of the virus in the internal tissues (prevalence of 10.2%; 71/695) exclusively, followed by individuals with infection in gill only and individuals with infection in both tissues (Table 1). This pattern however was only significantly different at the high shore at Carlingford Lough (*χ*^2^ = 12.43, *df* = 2, *P* < 0.01) and not at the high shore at Dungarvan (*χ*^2^ = 2.29, *df* = 2, *P* = 0.32) and at the trestles at Carlingford Lough (*χ*^2^ = 1, *df* = 2, *P* = 0.61) (Table 1).

**Table 1:** PCR prevalence of OsHV-1 μVar in *Cerastoderma edule* tissue groups at the oyster culture sites

	Trestle			High shore		
	Gill	Internal	Gill & Internal	Gill	Internal	Gill & Internal
Dungarvan	–	–	–	6.4% (16/250)	*9.6%* (24/250)	6.4% (16/250)
Carlingford Lough	6.6% (15/227)	*8.8%* (20/227)	6.6% (15/227)	5.0% (11/218)	*12.3%* (27/218)	4.1% (9/218)

*Note*: Values in italic font represent the highest OsHV-1 μVar prevalence in cockles

**Fig. 3:**
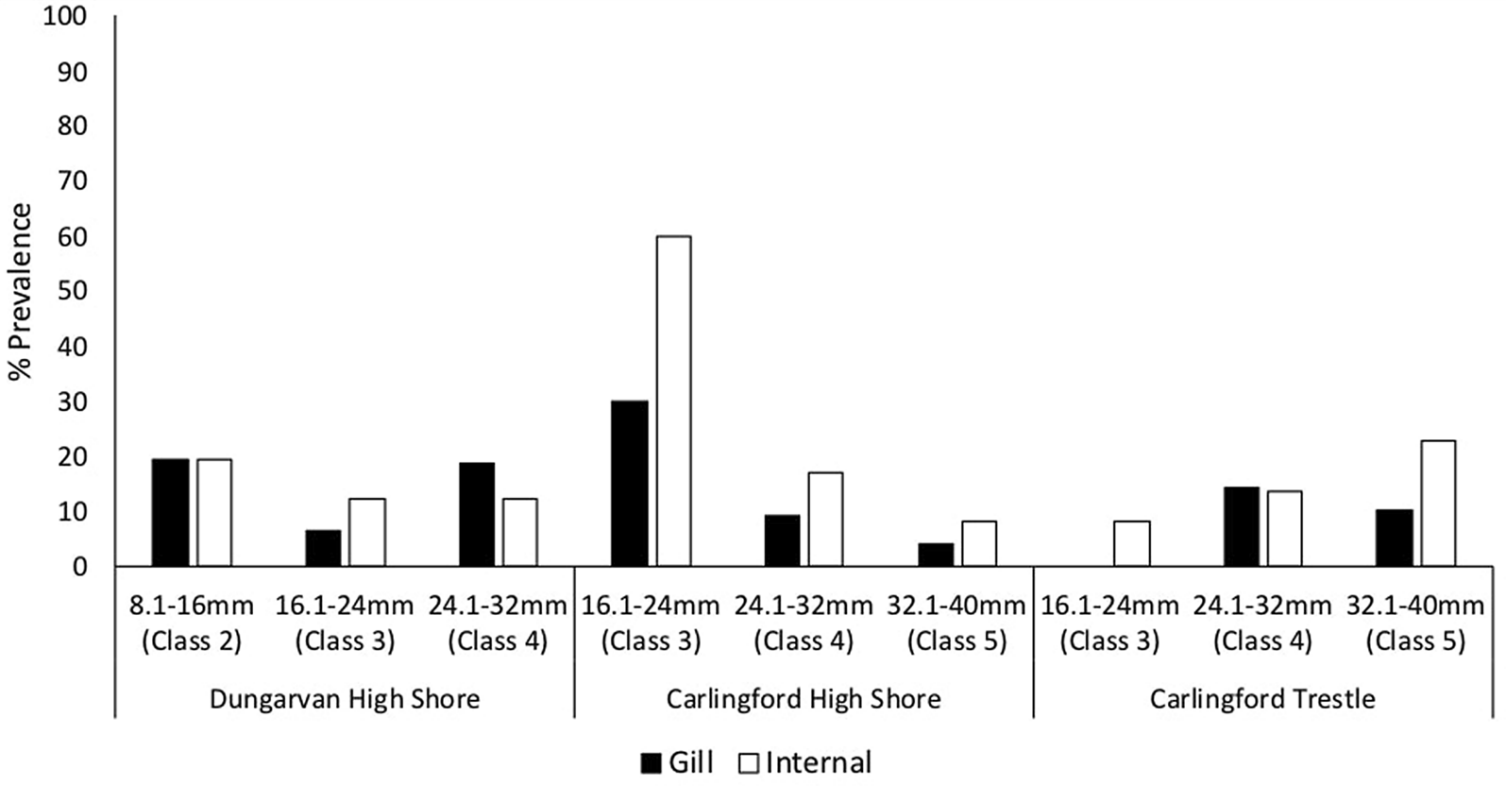
OsHV-1 μVar prevalence (%) in the *Cerastoderma edule* size classes at the study sites

Significant differences in prevalence between months were observed for both Dungarvan (*χ*^2^ = 25.15, *df* = 4, *P* < 0.01) and Carlingford Lough (*χ*^2^ = 17.95, *df* = 4, *P* < 0.01) with highest prevalence in May and lowest prevalence in April at both sites (Fig. 2). No significant trends were observed for herpesvirus prevalence and the size and age of the cockles (indicated by growth rings), with the virus being detected in all length and weight classes and all shell ring classes (Fig. 3).

**Fig. 4:**
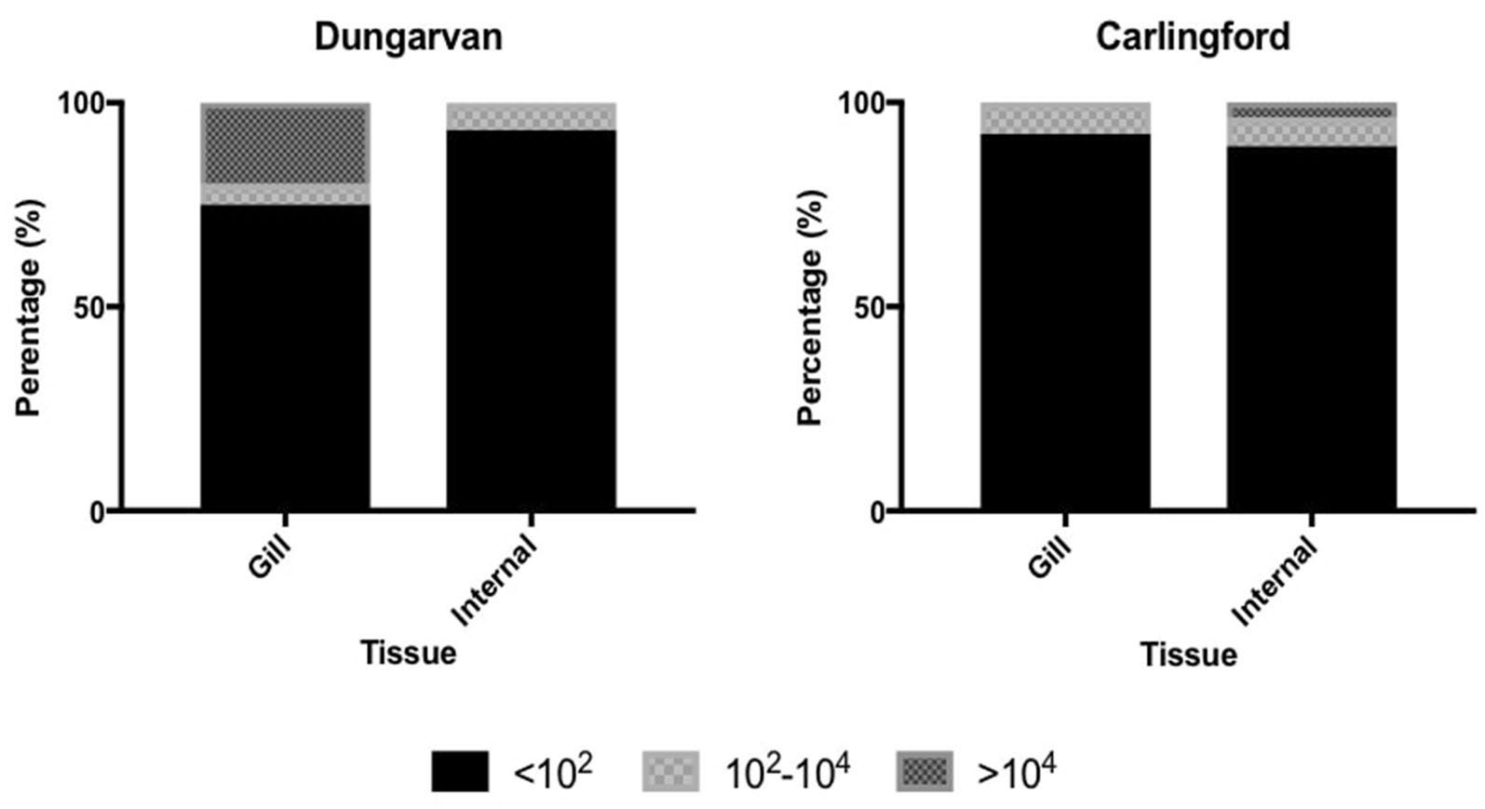
Mean OsHV-1 μVar loads in the gill and internal tissues of *Cerastoderma edule* at both study sites

**Fig. 5:**
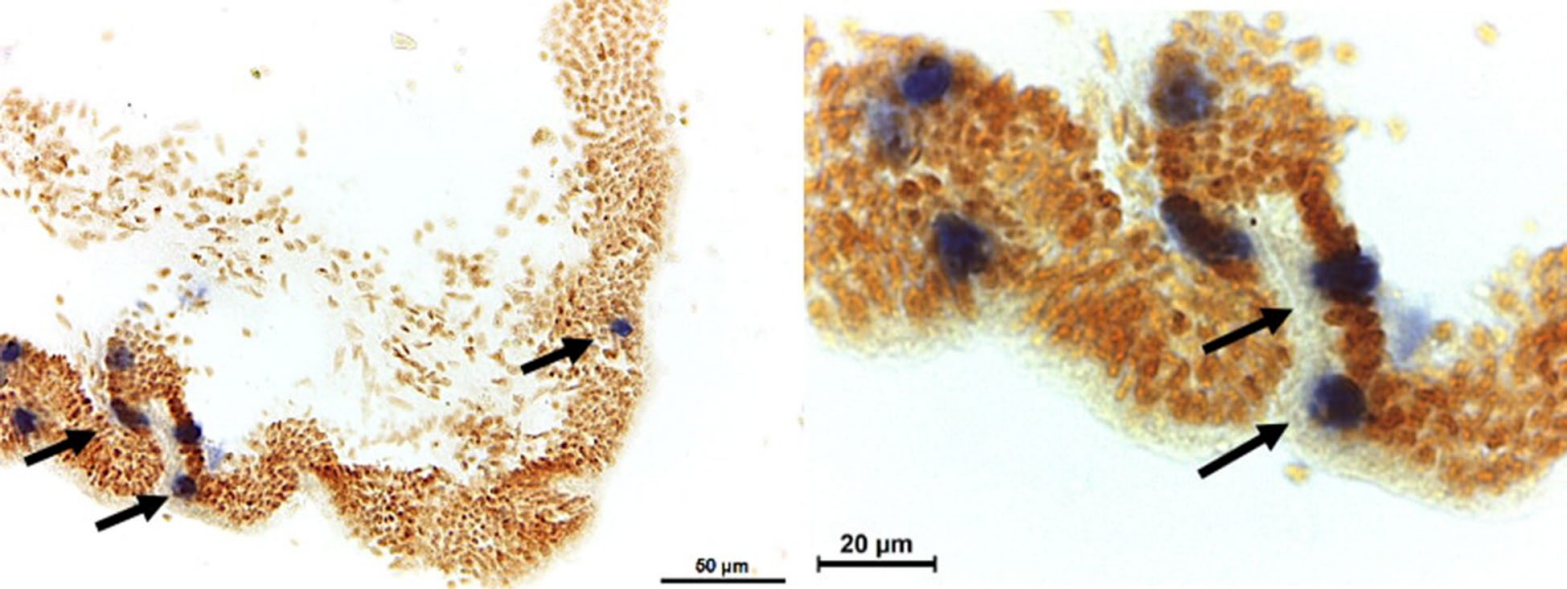
ISH staining of OsHV-1 μVar infected blood cells (dark blue) in cockle digestive epithelia

Viral load in cockle tissues was generally low with the majority of cockles sampled having less than 10^2^ and a small number having 10^3^ and 10^4^ viral copies (Fig. 4). Confirmation of the presence of OsHV-1μVar was carried out using ISH; a positive signal was observed for a small number of *C. edule* (83.3%; 5/6) screened, these had been collected in the field, for the laboratory trial (Fig. 5).

After sequencing of the PCR products of *C. edule* (*n* = 5), BLASTn analysis showed a match with OsHV-1 μVar for the sequence of the PCR-amplified products of *C. edule* with an average of 92.6% (88–95%) query coverage and 99% maximum identity to OsHV-1 μVar (GenBank: KU861511.1).

### Laboratory cohabitation transmission trial

Transmission was effected from cockles infected with OsHV-1μVar to naïve uninfected oysters in the laboratory. Prior to the trial commencing (collected in October 2015), 30 individuals of each species (cockles and oysters) were screened. Naïve *C. gigas* were uninfected with OsHV-1 μVar as expected. The initial sample of *C. edule* had a prevalence of 6.7% (*n* = 2/30) with a mean viral load of 1.1 × 10^1^ copies/μl of genomic DNA (range: < 10–3.0 × 10^1^ viral copies/μl of genomic DNA). Overall, the prevalence of infection in the *C. edule* held with the *C. gigas* in a single experimental tank was 3.2% (*n* = 2/63) with an average viral DNA load of 1.2 × 10^1^ copies/μl (range: < 10–1.2 × 10^4^ viral copies/μl of genomic DNA). OsHV-1 μVar was detected in a single dead oyster on Days 5, 8, 11 and 13. *C. gigas* held with *C. edule* in that single experimental tank had an overall OsHV-1 μVar prevalence of 4.4% (*n* = 4/90) with an average viral load of 3.0 × 10^3^ DNA viral copies/ μl of genomic DNA (range: < 10–1.2 × 10^4^ viral copies/μl of genomic DNA). No OsHV-1 μVar was observed in the *C. edule* or *C. gigas* in the two additional experimental tanks/replicates.

**Fig. 6:**
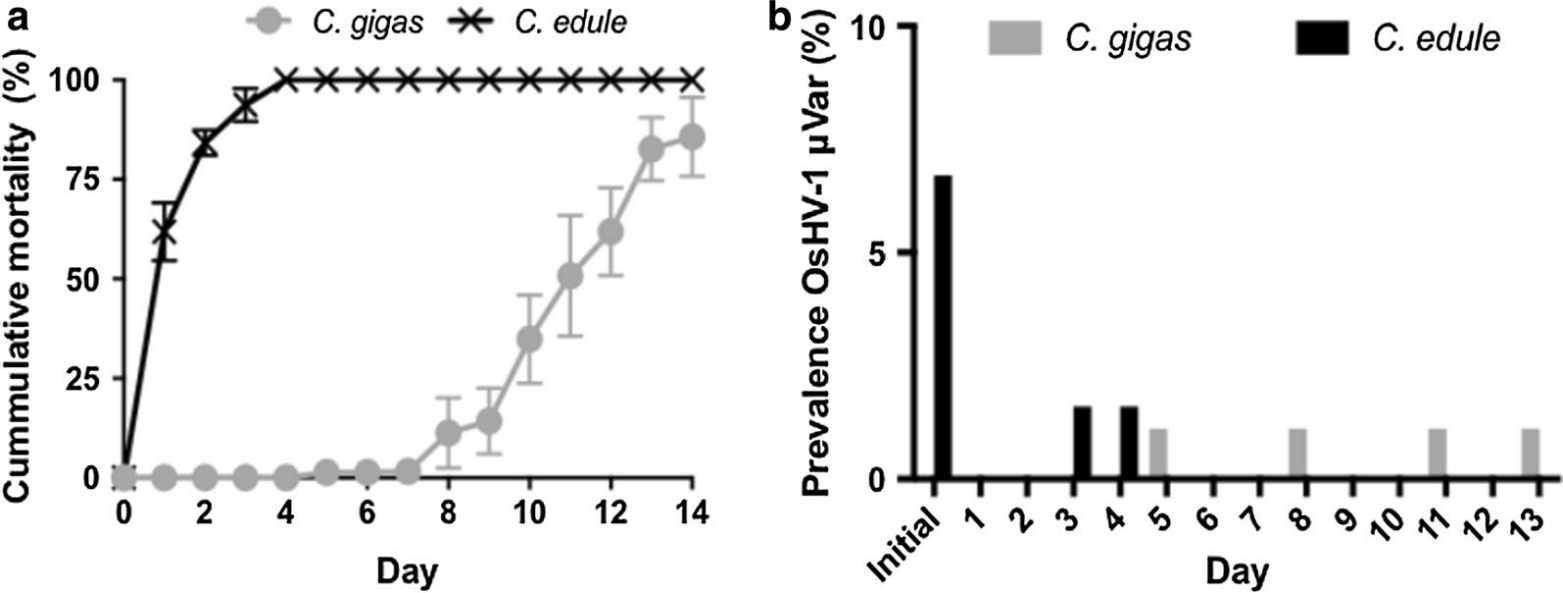
Cumulative mortality (**a**) and OsHV-1 μVar prevalence (%) (**b**) in the laboratory trial

During the laboratory trial, cumulative mortality of up to 100% was observed in *C. edule* in all three replicate experimental tanks within the first 4 days of the trial (*n* = 63). *Crassostrea gigas* showed low mortality up to Day 7 in all three experimental tanks (< 10%), after Day 7 mortality increased up to 66.7–100% (with a mean of 85.7% for all three experimental tanks) at the end of the trial (Fig. 6). All *C. gigas* in the control tank were still alive at the end of the trial.

## Discussion

In this study, the potential for an intertidal ecosystem engineer *C. edule* to become infected with and to act as an alternate host of OsHV-1 μVar was determined, through field screening, laboratory transmission trials and using a variety of diagnostic methods. The field survey demonstrated the ability of a virus to jump host to an alternate infaunal bivalve with potential further transmission through an ecosystem and potentially greater geographic dispersal via trophic food webs, as was demonstrated by Bookelaar et al. [42]. In that study, OsHV-1 μVar was detected in *C. maenas*, a predator/scavenger of *C. gigas* and *C. edule* [52], and transmission of the virus occurred between infected crabs and naïve *C. gigas*. This suggests that OsHV-1 μVar is exhibiting plasticity, by spreading to other bivalves. Detection of OsHV-1 μVar did not show a significant correlation with cockle size, weight and age (number of shell rings), which indicated that all cockle cohorts were likely to be infected with the virus. In addition, the laboratory trial indicated that cockles can transmit viable OsHV-1 μVar back to oysters within five days post-exposure and at a low temperature of 14 ℃, below the temperature (16 ℃) considered necessary for activation and replication of the virus. The mortality of all *C. edule* by the fourth day of the trial in all three experimental tanks (one with infected *C. edule* and two with uninfected *C. edule*) was not exclusively due to OsHV-1 μVar but possibly due to the artificial holding conditions or another health condition. *Cerastoderma edule* typically do not perform well (i.e. survive) in artificial holding conditions in particular stand-alone tanks (our unpublished data). Additionally, a substrate was not provided, which may have acted as a stressor by preventing their normal burrowing behaviour. This was a deliberate step to encourage the *C. edule*, if infected, to shed the virus into the tank system holding the *C. gigas*.

Herpesvirus infection was detected in the oysters being cultured at the sites in this study, but at a lower level compared to previous years in Ireland [35, 45, 53]. In this study, oysters selectively bred for resistance to OsHV-1 μVar were used (oyster farmers, personal communications), which might be the main explanation for the lower prevalence in oysters. Moreover, temperatures were lower compared with previous years and rarely passed the temperature level (16 ℃) considered necessary for OsHV-1 μVar to activate, but the subsequent laboratory trial demonstrated the transmission could occur at 14 ℃ from cockles to oysters. Survival of aquatic viruses such as Abalone herpes virus outside their natural hosts, has been mostly observed at lower temperatures [54]. In addition, a previous study investigating prevalence of OsHV-1 in *Ostrea edulis* observed a lower optimal temperature of 6–12 ℃ for *O. edulis*, compared to the threshold temperature of 16 ℃ in *C. gigas* [55]. In addition, a 14-fold OsHV-1 μVar replication was observed in mussels *Mytilus* spp. at 13 ℃ under experimental conditions [41]. The findings of this study would suggest that OsHV-1 μVar may be opportunistic, adapting to new circumstances, such as fewer available susceptible oysters due to the use of selectively bred oyster stocks more resistant to the virus, resulting in a “species jump” to another cohabiting bivalve species, and being transmissible at lower seawater temperatures.

A high percentage of the cockles showed lower viral copies than the viral copy number of 10^4^ mg^−1^ associated with oyster mortalities [56]. This phenomenon was also found in a recent Australian study where low viral copies of OsHV-1 μVar were detected in other marine invertebrates; mussels, whelks and barnacles and it was suggested that these species could function as reservoirs and/or support transmission of OsHV-1 μVar in the environment [39]. However, that study did not investigate if those infected invertebrates could transmit the virus to oysters. Of significance in this study, a few individuals did have a viral copy number of 10^4^ mg^−1^ cockle tissue, which would indicate replication of the virus in the cockles.

## Conclusions

As a possible consequence of this ‘species jump’, and finding the virus in all surfaced cockle sizes/ages, it is likely infected cockles could be consumed by mobile top predators more readily, like bird and fish species, facilitating the transmission and greater geographical dispersal of the virus and potentially transmitting the virus to new carriers, reservoirs or possibly new hosts. The potential of OsHV-1 μVar to extend its geographical range and be introduced to uninfected marine habitats is possible with the migration of such mobile predators. Certainly, previous studies indicated that birds contribute to geographical jumps of emerging infectious diseases and have been proven carriers of Lyme disease and influenza A [57] and white spot disease in penaeid shrimps *Penaeus monodon* [58]. Changes in environmental conditions, in particular warming seas, are predicted to create a more conducive environment for viral replication. Such changes might result in significant cockle mortalities and as a keystone species any negative impacts through increased mortality or reduced physiological ability are likely to have wider ecosystem and commercial impacts.

## Data Availability

Data supporting the conclusions of this article are included within the article. The datasets generated and analysed during the present study are available from the corresponding author upon reasonable request.
